# Drug Resistance Pattern of MTB Isolates from PTB Patients

**DOI:** 10.1155/2013/862530

**Published:** 2013-10-24

**Authors:** Rajani Ranganath, Vijay G. S. Kumar, Ravi Ranganath, Gangadhar Goud, Veerabhadra Javali

**Affiliations:** ^1^Department of Microbiology, Navodaya Medical College and Research Centre, Raichur, Karnataka, India; ^2^Department of Microbiology, JSS Medical College and Research Centre, Mysore, Karnataka 570 015, India; ^3^Usha Kidney Care, Bellary, Karnataka 583103, India; ^4^Department of Community Medicine, VIMS, Bellary, Karnataka 583104, India; ^5^Department of Orthopaedics, Navodaya Medical College and Research Centre, Raichur, Tamil Nadu 641043, India

## Abstract

*Background*. TB is a global pandemic disease. All TB control programs were not successful due to the emergence of multidrug resistance in *M. tuberculosis* strains. Objective of the present study was to detect the rate of MDR-MTB in this part of India. *Methods*. One hundred and thirty clinical MTB strains isolated from patients on treatment and confirmed as MTB by MPT64 antigen detection were tested for drug susceptibility against Streptomycin, INH, Rifampicin, and Ethambutol by MBBact automated system. *Result*. Thirty-two were MDRs (25.61%). 31.2%, 28%, 17.6%, and 21.6% were resistant to INH, RIF, Ethambutol, and Streptomycin, respectively. Resistance to either INH or Rifampicin was 20.8% and 13.88%, respectively. Combined INH and Rifampicin resistance was seen in 18.05% isolates. *Conclusion*. Drug resistance rate is high in patients treated previously and who have been irregular on treatment.

## 1. Introduction

Tuberculosis (TB) is the second leading cause of death from an infectious disease worldwide after human immunodeficiency virus (HIV). Inspite of free supply of drugs, 1.4 million TB deaths occurred worldwide in 2011. Recently, World Health Organization has estimated that 3.7% of new TB cases are MDRs. MDR-TB global average rate is 20%. About 9% of these cases also are resistant to at least one injectable second line antitubercular drugs. These strains are called extensively drug resistant (XDR) TB cases [1]. During the middle of twentieth century, tuberculosis rate in Europe and North America decreased to an extent that it was thought as totally eradicated. Health care providers started to announce that TB is eradicated. TB sanatoriums were closed. But M. tuberculosis bounced back in 1980s with a vengeance and has spread all over the world. Unholy nexus between TB and HIV has further increased not only TB rate but also mortality. Drug resistance (DR) in MTB is a manmade problem. Defaulting by the patient, poor quality of drugs and lack of awareness have contributed to the present grim situation of TB management. In 1993, increasing reports of MDRTB were noted from USA [2] and WHO declared TB as global emergency [3].

WHO's millennium development goal to reduce TB by 2015 has failed. Drug resistance in MTB is manmade and is a consequence of suboptimal regimens and treatment interruptions [4]. MTB strains exhibiting resistance to INH and Rifampicin, the two main first line drugs, are designated as MDRTB strains. These MDR strains require prolonged treatment using second line drugs which are highly toxic and less effective [5, 6]. WHO and International Union against TB and Lung diseases (IUATLD) initiated a global TB surveillance program in 1990 to take stock of global prevalence of drug resistant TB opened global surveillance centres as state referral laboratories (TB SRLs) [7]. The responsibility of these centres was MDRTB case DATA collection Drug resistance in MTB is of two types, primary or Innate resistance and secondary or Acquired resistance. Acquired drug resistance is due to many reasons, defaulting by the patient is one reason. Presence of primary resistance and acquired resistance in MTB strains is an indicator of TB control program efficacy (of the past and the ongoing). Distribution and rate of MDR and XDR-TB are not uniform. They vary in different places, regions, and countries. This is the first study attempting to detect in vitro drug resistance pattern of MTB isolates covering a population of ten millions using MBBacT automated culture and DST system.

## 2. Materials and Methods

### 2.1. Study Setting

This study was done at JSS teaching hospital, Mysore, Karnataka, which is a referral centre for carrying out DST, from April 2011 to May 2012.

### 2.2. Study Population

130 sputum smear positive cases aged 20 years and above were included and cases below the age of 20 years and sputum smear negative cases were excluded. All these 130 cases were previously treated for TB and then referred to JSS centre for mycobacterium culture and DST. These mainly included patients who had been treated at this centre or other health centres from neighboring states implementing directly observed treatment under supervision (DOTS) strategy or patients who are referred by private practitioners. Most of private practitioners do not follow RNTCP guidelines for DST and hence patients who had relapsed or defaulted (with a history of previous treatment) or who had remained sputum positive after ≥5 mths of ATT were referred for DST. About 104 (80%) of the cases were failures, 13 (10%) were relapse, and remaining 13 (10%) were treatment after default. Sputum samples were collected from patients before starting retreatment.

Early morning expectorated sputum samples were collected in sterile containers on two successive days. Smears were made from the collected samples and stained by Ziehl Neelsen (ZN) staining. The smears were screened for acid fast bacilli (AFB) and positive smears were graded as per Revised National Tuberculosis Control Programme (RNTCP) guidelines [8].

### 2.3. Processing of Sputum Specimens by Modified Petroff's Method

5 mL of sputum specimen was transferred to centrifuge tube and double the volume of sterile 4% sodium hydroxide (NAOH) was added aseptically. It was mixed well and placed in incubator at 37°C for 15 mins. After 20 mins, centrifuge tube was removed from incubator and 15 mL of sterile distilled water (SDW) was added. It was then centrifuged at 3000 ×g for 15 mins. Supernatant fluid was discarded slowly into a container with 5% phenol solution. Pellet was washed with SDW at 3000 ×g for 15 min and supernatant was discarded. Sediment was later used to inoculate into MBBacT culture bottle.

### 2.4. MBBacT Automated System

Principle: if microorganisms are present in the test sample, CO_2_ is produced as organisms metabolize substrates in culture medium. Colour of gas permeable sensor installed at the bottom of each culture bottle changed to light green/yellow. 

### 2.5. MP Culture Bottle

It contains 10 mL of media and an internal sensor that detects CO_2_ as an indicator of microbial growth.

### 2.6. MBBacT Antibiotic Supplement Kit

It consists of antibiotic supplement and reconstitution fluid. antibiotic supplement was reconstituted with 10 mL of Reconstitution fluid before use. Once reconstituted, it had a shelf life of 7 days. Reconstitution fluid contains components which ensure optimal growth of *Mycobacteria* present in sputum samples. MBBacT culture bottles and antibiotic supplement kit was stored under refrigeration (2–8°C) and was equilibrated to room temperature (30 mins) before use. 

### 2.7. Inoculation

MP culture bottle was disinfected with alcohol pad and was allowed to dry. Aseptically 0.5 mL of reconstituted antibiotic supplement was added to each of culture bottle. Pink tint in medium indicated that reconstitution fluid has been added successfully. 0.5 mL of NAOH decontaminated sputum sample was inoculated and loaded into instrument.

### 2.8. Interpretation

When instrument indicated a positive bottle by flagging, ZN smear was done to detect AFB. If *nonmycobacterial* organisms were seen on grams stain, entire bottle contents were reprocessed through another decontamination procedure and inoculated into a new culture bottle or discarded and another specimen for culture was obtained. Bottles flagged negative only after 42 days. Inoculated MBBacT culture bottles were autoclaved before disposal. 

### 2.9. Identification of MTB

Identification of MTB was done by MPT64 immunochromatographic antigen detection test, manufactured by SD Bioline, South Korea [9].

### 2.10. SD Bioline TB Ag MPT64

Mouse monoclonal anti-MPT64 was immobilized on the nitrocellulose membrane as the capture material (test line). Colloidal gold particles were used for antigen capture and detected in a sandwich type assay. Presence of only control band indicates negative result. Presence of 2 coloured bands within the result window, no matter which band appears first, indicates a positive result. If the control band is not visible, test is invalid [9].

### 2.11. DST in MBBacT

Processing for AST: proportion method was used for determining DST as per RNTCP guidelines [8]. MTB H37Rv strain was used as control strain. Six process bottles were required for each MBBacT positive sample. Four bottles were labeled as S, I, R, E (Streptomycin, Isoniazid, Rifampicin, and Ethambutol). One bottle was labeled as Direct Control (DC) and other as Proportional Control (PC). 0.5 mL of restoring fluid (contains nutrients to enhance the growth of *mycobacteria*) was added to all six bottles aseptically. 0.5 mL of corresponding antibiotics were added to the process bottles labeled as S, I, R, and E aseptically. 0.5 mL of sterile distilled was added in Direct Control and Proportional Control aseptically.

Source of inoculum: 0.5 mL of flagged MBBacT positive culture bottle was called seed bottle. This “seed” bottle was taken for AST processing. 

All drugs were in chemically pure powder form and were stored at −20°C in desiccators as recommended by manufacturer (HiMedia). Drug concentrations used were INH 0.2 mg/L, RIF 40 mg/L, EMB 2 mg/L, and SM 3 mg/L and were dissolved in deionized water. All stock solutions were sterilized by membrane filtration through 0.22 *μ*m pore sizes and stored at −80°C in small aliquots. The frozen drug solutions were used immediately after thawing and the remaining was discarded and never stored in freezer again. Working solution was prepared freshly from the stock solution.

### 2.12. Preparation of Inoculum for Drug Containing Bottles and Direct Control

Seed bottle was vortexed to break the clumps. 3 mL of inoculum from this bottle was withdrawn into a sterile bijou bottle with 2-3 mm glass beads. It was vortexed to break the clumps and was standardized to 1Mac Farland. 0.5 mL of the standardized inoculum was added to the process bottles labeled as S, I, R, and E and Direct Control aseptically.

### 2.13. Preparation of Inoculum for Proportional Control

0.1 mL of inoculum was taken from positive MBBacT bottle and added to 9.9 mL of sterile distilled water (1/100 dilution). 0.5 mL of inoculum from this was added to the Proportional Control bottle aseptically. Bottles were loaded into MBBacT.

### 2.14. Reading and Interpretation of Results

When the instrument indicated a positive bottle, bottles were removed according to the procedure. The test was invalid if the DC and PC were not flagged positive within 15 days. If flagged positive within 2 days or less, then contamination or a fast growing AFB was suspected so that the bottles were subcultured and the tests were repeated if required. If DC and PC were determined positive within 1 day of each other then it indicated that 1/100 dilution was not done properly.

If the antibiotic containing bottles were flagged positive after the PC bottle, then the test organism was considered as susceptible provided with DC bottle flagged positive. If the antibiotic containing bottles were flagged positive before the PC bottle, then the test organism was considered as resistant provided with DC bottle flagged positive.

Data entry and analysis were performed using SPSS version 17. Chi square and percentages were applied wherever necessary. *P* value < 0.05 was considered as statistically significant. The research proposal was cleared by medical faculty ethical review committee.

## 3. Results

Out of 130 sputum smear positive cases, 78 (60%) were male and 52 (40%) were female. Among 130 isolates, 1 (0.76%) was contaminated and 1 (0.76%) did not yield growth of *mycobacterium*. Average turnaround time for culture by MBBacT method was 5 to 7 days. All remaining 128 isolates were identified as MTB by MPT64 antigen detection test and were put up for DST against SIRE drugs. DST results were invalid in 3 out of 128 isolates.

Table 1: among remaining 125 isolates, 72 (57.6%) were resistant to one or more drugs. Resistance to INH, RIF, EMB, and SM was found to be 31.2%, 28%, 17.6%, and 21.6%, respectively (*P* < 0.05, CI 95%).

Table 2 shows the resistance pattern of 72 drug resistant isolates. Single drug resistance was observed in 31 (25.61%), any two drug resistance in 22 (18.18%), any three drug resistance in 14 (11.57%), and all four drug resistance was found in 5 (4.13%). MDR was found in 32 (25.61%). Among monoresistance INH 15 (20.8%) was found to be the highest proportion, followed by RIF 10 (13.88%), and in polyresistant strains, the highest proportion was found in INH + RIF combination 13 (18.05%) (*P* value < 0.05, CI 95%).

## 4. Discussion

Drug resistant tuberculosis is either acquired due to poor management of treatment or transmission from infectious drug resistant TB patients. As found in many other studies history of antitubercular treatment has been consistently associated with risk of MDR-TB [9]. 

Overall MDR rate observed in this study is 32 (25.61%). Our findings are concordant with other studies reported from Chandigarh (27.6%) [10], Tamil Nadu state (25%) [11], Mumbai (25.25%) [12], and Gujarat (30.2%) [13]. But higher rates were observed in Dehradun (57.22%) [14] and Delhi 53.6% [15] and the lowest rates were seen in Sewagram Wardha (9.2–9.6%) [16, 17]. High rate of MDRTB in our setting is understandable, as this is a referral centre for mycobacterial culture and DST and therefore receives a large number of samples from chronic patients.

The highest resistance is seen in Isoniazid (31.2%) which is the most popular drug followed by Rifampicin (28%), Streptomycin (21.6%), and Ethambutol (17.6%). Similar resistance pattern was reported by Vijay et al. in Bangalore to INH (27.4%), RIF (15.5%), SM (23%), and EMB (6.6%) [18]. Resistance pattern reported by Sethi et al. from Chandigarh in previously treated patients to INH (46.9%), RIF (27.6), SM (22.22%), and EMB (10%) [10]. Resistance reported by Lina et al. from Mumbai in previously treated patients shows INH (30.41%), RIF (58.55%), SM (46.95%), and EMB (3.67%) [12]. 

Resistance to INH was found to be 31.2% in this study. Similar findings were reported by Vijay et al. from Bangalore (27.4%) [18] and Dam et al. from Delhi (20.18%) [19]. Still higher rates of INH resistance were reported by Paramasivan et al. from Tamil Nadu (81%) [20], Sethi et al. from Chandigarh (46.9%) [10], and Gopi et al. from Raichur (52.3%) [21]. This high resistance may be because of poor compliance by the patients and its widespread use in treatment of tuberculosis.

 Rifampicin resistance in our study is 28%. Resistance for RIF is considered as surrogate marker for detection of MDR-TB. Similar findings were reported by Paramasivan et al. from Tamil Nadu (25%) [11], Sethi et al. from Chandigarh (27.6%) [10], and Jain et al. from New Delhi (33.3%) [17]. Still higher rates of RIF resistance are reported by Rawat et al. from Uttarakhand (57.22%), [14], Janmeja and Raj from Haryana (49%) [22], and Paramasivan et al. from Raichur (100%) [20]. Reason for this high resistance may be due to irregular use in other conditions like leprosy, pyrexia of unknown origin (PUO), and leishmaniasis.

In this study drug resistance against streptomycin was found to be 21.6%, which is in concordance with reports by Vijay et al. from Bangalore (23%) [18] and Rawat et al. from Uttarakhand (22.22%) [14]. Resistance of 17.6% was noted against Ethambutol in our study. Similar corroborative resistance pattern was seen in studies from Negi et al. from Delhi (20.65%) [23] and Rawat et al. from Uttarakhand (10%) [14].

In this study INH monoresistance (20.8%) was found to be high. Our results are concordant with reports by Sethi et al. from Chandigarh (14.3%) [10] and Ramachandran et al. from Gujarat (11.7%) [24]. This may be due to INH prophylactic therapy (IPT) given to patients without ruling out active TB among HIV positives. IPT can increase chances of drug resistant TB [25]. 

Inefficiency in TB control programmes and irregular antitubercular drug usage leading to accumulation and multiplication of resistant strains is supported by remarkable increase in drug resistance among retreated cases. Because of selection bias in hospital, lack of comorbidity and HIV status and robust retrospective data has limited our study. 

One most important limitation of this study is previous treatment histories, demographics, and other data like IPT, and information about second line drug susceptibility like quinolones were not available for analysis, restricting our ability to derive concrete conclusions. Other limitations are data as they are not representative of the whole community and are limited to one hospital. A community based multicenter study, which includes all parts of the country and uses the full spectrum of drugs, is needed to describe the true prevalence of MDRTB in India.

## 5. Conclusion

The major problem in the treatment of pulmonary tuberculosis is multidrug resistance. Emergence of MDRTB can be reduced by detecting the drug resistance pattern and by treating with the second line antituberculous drugs in appropriate regimens in failure and relapse cases. MDR rate in our study was significantly higher among treatment failures compared to relapses and treatment after default cases, underlying the need for early identification of treatment failure by early referral for culture and drug susceptibility testing and initiation of appropriate treatment.

Emphasis should be laid on prompt case detection, routine and quality assured DST facilities for high risk patients, prompt administration of anti-TB drugs, and strengthening the coordination of nongovernmental organizations (NGOs) and private practitioners as per the guidelines laid down by RNTCP. Additional studies detecting the drug resistance pattern is the need of the hour to delineate risk factors and to formulate plans for future management of tuberculosis in high MDRTB settings. 

## Figures and Tables

**Table 1:** Sensitivity pattern of MTB to four antitubercular (ATT) drugs.

Name of the drug	No. of sensitive strains (%)	No. of resistant strains (%)
Isoniazid (INH)	86 (68.8)	39 (31.2)
Rifampicin (RIF)	90 (72)	35 (28)
Ethambutol (EMB)	103 (82.4)	22 (17.6)
Streptomycin (SM)	98 (78.4)	27 (21.6)
Resistance to any drug	—	72 (57.6)

**Table 2:** Resistance pattern of 72 drug resistant strains of MTB to four ATT drugs.

Number of drugs	Name of drugs	No. of resistant strains (%)	Total (%)
1 drug	Isoniazid (INH)	15 (20.8)	31 (25.61)
	Rifampicin (RIF)	10 (13.88)	
	Ethambutol (EMB)	4 (5.55)	
	Streptomycin (SM)	2 (2.77)	

2 drugs	INH + RIF	13 (18.05)	22 (18.18)
	INH + SM	4 (5.55)	
	INH + EMB	3 (4.16)	
	EMB + SM	2 (2.77)	

3 drugs	INH + RIF + SM	9 (12.5)	14 (11.57)
	INH + RIF + EMB	5 (6.94)	

4 drugs	INH + RIF + SM + EMB	5 (6.94)	5 (4.13)

MDR-TB		32 (25.61)	
